# Differential protein profiling as a potential multi-marker approach for obese patients with heart failure: A retrospective study

**DOI:** 10.1038/s41598-018-26118-9

**Published:** 2018-05-21

**Authors:** Andrei Timotin, Mathieu Cinato, Frederic Boal, Sebastien Dejean, Rodica Anesia, Oleg Arnaut, Christine Lagente, Jerome Roncalli, Franck Desmoulin, Helene Tronchere, Oksana Kunduzova

**Affiliations:** 1 INSERM U1048, University of Toulouse, Toulouse, Cedex 4 31432 France; 2 Toulouse Mathematics Institute, University of Toulouse, Toulouse, Cedex 4 France; 3 Department of Cardiology, Toulouse University Hospital, Toulouse, Cedex 9 France; 4 Toulouse NeuroImaging Center, ToNIC, University of Toulouse, Inserm U1214, Toulouse, Cedex 3 France

## Abstract

Identification of novel circulating biomarkers predicting death and major cardio-metabolic events in obese patients with heart failure (HF) remains a research priority. In this study, we compared multi-marker profile of non-obese (NOB) and obese (OB) HF patients in relation to mortality outcome. The new multiplex proximity extension assay technology was used to analyze the levels of 92 proteins in plasma samples from HF patients according to body mass index (BMI) categories. At 2*-*year follow*-*up, all-cause mortality rates were significantly greater in NOB patients (BMI < 30 kg/m^2^) compared to the OB patients (BMI > 30 kg/m^2^) with HF (odds ratio 26; 95% CI: 1.14–624, p < 0,04). Quantitative proteomic analysis revealed thirteen distinct proteins expression profiles of OB and NOB HF patients. Among these proteins, RAGE, CXCL6, CXCL1, CD40, NEMO, VEGF-A, KLK6, PECAM1, PAR1, MMP1, BNP and NTproBNP were down-regulated, whereas leptin was up-regulated in OB HF patients. In addition, an inverse correlation between plasma BNP levels and leptin in OB HF patients was observed (*r* = −0.58 p = 0.02). This study identifies specific plasma protein signature in OB and NOB patients with HF in relation to mortality outcome.

## Introduction

The co-occurring of obesity and heart failure (HF) is one of the key issues and critical challenges in clinical practice. Several cohort studies have shown a relationship between elevated body mass index (BMI) and chronic medical conditions such as diabetes mellitus, hypertension, hyperlipidemia, coronary artery disease, HF and stroke^1–3^. A rational assumption that overweight or obesity is associated with higher mortality than that of normal weight, however, is not conclusive. Longitudinal studies have shown the existence of an “obesity paradox”, a clinical phenomenon in which obese (OB) persons have a lower risk of mortality or better survival within clinical subpopulations^4^. This paradoxical benefit of obesity has been shown to exist for a wide range of cardiovascular diseases (CVD)—myocardial infarction, hypertension, aortic stenosis, patients who have had a coronary bypass, atrial fibrillation, and patients with cardiac implants^5^. This paradox also exists in patients with cancer, renal disease, chronic obstructive pulmonary disease, pneumonia, stroke, chronic respiratory insufficiency, and diabetes mellitus^6^. The obesity paradox has been specifically demonstrated in HF patients with a consistency of results seen among a wide range of clinical subpopulations across geographical locations, gender, age range, and the presence or absence of comorbidities^7^. Determining whether the obesity paradox is a causal phenomenon among patients with HF is clinically relevant and mechanisms related to the increased risk of HF and the associated effects once HF is diagnosed remain a subject of fierce scientific debate.

In clinical practice, the diagnosis and therapeutic strategy of HF in OB patients is based on relatively subjective assessments of diverse symptoms representing multiple endophenotypes. Indeed, obesity is a heterogeneous condition and a true reflection of the impact of BMI variability on cardiovascular health can only be captured by subdividing obesity into phenotype. Phenotyping beyond BMI should not be limited to anatomical or physiological variables. The dynamic molecular profiling of HF patients in relation to BMI-based approaches can also help with diagnosis, treatment and preventive measures of OB patients, as well as stratify OB patients into more homogeneous, clinically distinct subpopulations. Currently there is a paucity of data regarding the differences in the biomarker profiles of non-obese (NOB) versus OB patients with HF. Clinical and preclinical studies have identified a number of circulating proteins that may serve as putative biomarkers for diagnosing and treating HF^8^. However, the utility of this panel of indicators to serve as clinically useful biomarkers in OB patients with HF is limited by a lack of data regarding unique cardiovascular signature in conditions combining obesity and HF^9^.

## Research Design and Methods

### Data

The data for the present study were collected in the period between May 2013 and June 2016 at the Rangueil University hospital, Toulouse, France. The diagnosis of HF was established according to current guidelines^10^. Patients had known stable HF with more than 3 months without any decompensation episodes, irrespective of clinical severity (stage II to IV of New York Heart Association (NYHA) classification) and etiology. The association of absolute BMI values with the 2-year mortality was investigated in HF patients. We retrospectively selected 16 male patients (mean age 63 ± 9 years) in order to compare plasma level of 92 proteins related to cardiovascular, inflammatory and metabolic status in NOB and OB HF phenotypes. Patients were assigned to two groups according to BMI and outcome parameter: NOB patients (BMI < 30 kg/m^2^, 63% of mortality rates, n = 8) and OB patients (BMI ≥ 30 kg/m^2^, 0% of mortality rates, n = 8). The study was approved by a local ethics committee and included only patients who provided written informed consent. The research protocol conforms to the ethical guidelines of the 1975 Declaration of Helsinki. The protocols of plasma collection were approved by the Institutional Research Board of INSERM and Toulouse University Hospital Ethics committee. Venous blood samples were drawn from the patients into EDTA-containing tubes and were centrifuged and stored at − 80 °C.

### Proximity extension immunoassay (PEA)

The plasma levels of 92 cardio-inflammatory-related proteins were simultaneously measured by a PEA using the Proseek Multiplex CVD kit I (Olink Bioscience, Sweden) as previously described^11^. Briefly, each protein is recognized by a pair of oligonucleotide-labelled antibodies and when binding to their correct targets, they give rise to reporter amplicons which are amplified and quantified by microfluidic-based real-time PCR (BioMark HD System, Fluidigm). The data obtained is normalized and used for the relative quantification of the concentration of each analyte^7^. The PEA approach offers the same level of performance as ELISA and comparable sensitivity to standard ELISA kits with much less sample and a higher dynamic range. A list of CVD measured proteins is presented in Supplementary Table S1.

### Data Analysis

Data analysis was performed by employing a pre-processing normalization procedure. For each data point, delta Cq (dCq) values were obtained by subtracting the value for the Extension control, thus normalizing each sample for technical variation within one run. Normalization between runs is then performed by subtraction of the Interplate Control (IPC) for each assay. In the final step of the pre-processing procedure the values are set relative to a fixed background level determined by Olink. The generated Normalized Protein Expression (NPX) unit is on a log2 scale where a larger number represents a higher protein level in the sample, typically with the background level at around zero, although it might differ between runs. Linearization of data is performed by the mathematical operation 2xNPX. Statistical analyses, e.g. coefficient of variation calculations were performed on linearized values.

### Statistical Methods

The summary statistics were presented by number (percentage), mean (SD) or median (IQR), as appropriate. The median duration of follow-up was calculated from the time of diagnosis of diabetes to the end of the follow-up. Non-parametric Kruskal–Wallis test or the chi-squared test was used to compare different study parameters across the BMI categories. The death rates and their 95% confidence intervals (CI) per 1000 person-years under different BMI categories were calculated using standard life-table analysis technique. Unpaired Student’s t-test was used for comparison of a protein concentration in plasma between NOB and OB HF patients.

## Results

**Figure 1:**
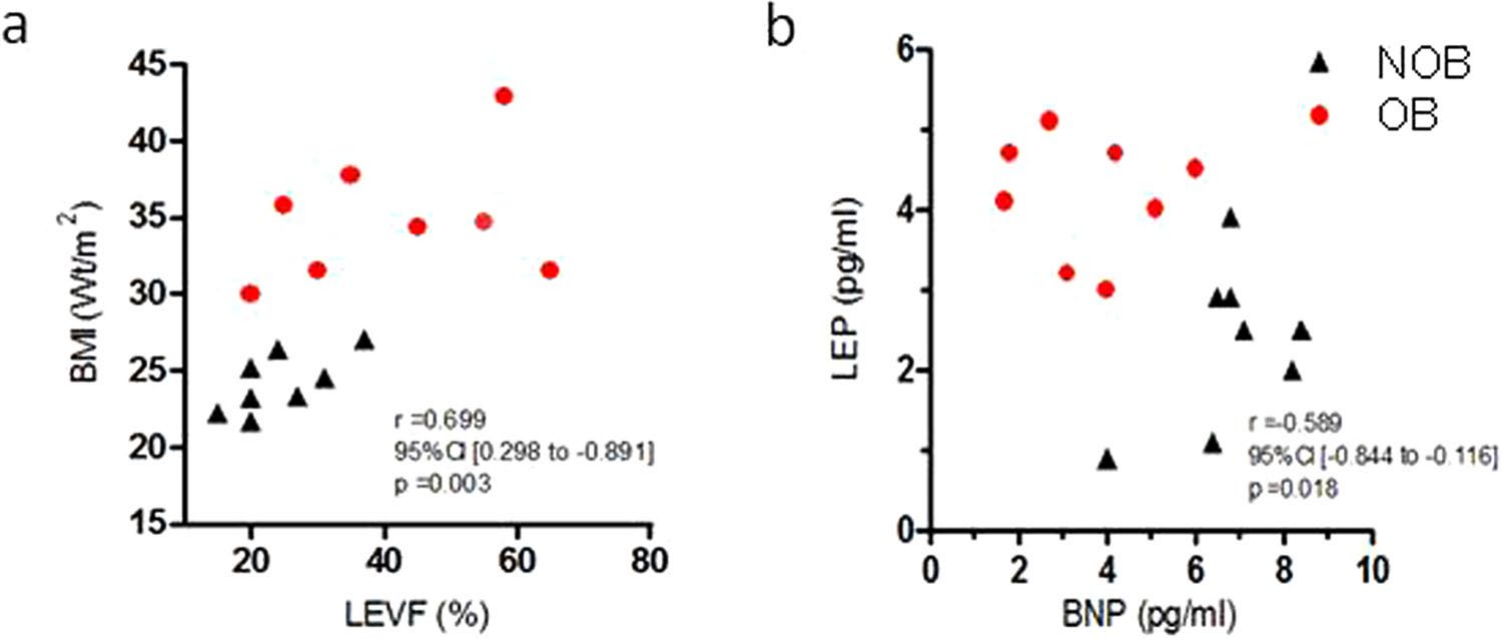
Correlation analysis of NOB and OB patients with HF. (**a**) Positive correlation between BMI and LVEF in HF patients (n = 16). (**b**) Negative correlation between plasma levels of BNP and leptin in NOB (n = 8) vs OB (n = 8) HF patients.

The comparison of demographic and clinical parameters between two groups revealed significant difference in Left Ventricular Ejection Fraction (LVEF) (p < 0.02), type 2 diabetes frequency (p < 0.01), and Brain Natriuretic Peptide (BNP) level (p < 0.05) (Table 1). As shown in Fig. 1a, a positive correlation between BMI and LVEF was found in the population (correlation coefficients Spearman’s *r* = 0.70 p < 0.01). Interestingly, a high BMI (>30 kg/m^2^) was associated with lower all-cause mortality rates at 2-year follow-up (odds ratio for death in NOB patients versus OB patients was 26 [95% CI, 1.14–624], p = 0.02). In addition, as shown in Fig. 1b, we identified an inverse correlation between plasma levels of BNP and leptin in OB HF patients (correlation coefficients Spearman’s *r* = −0.58 p = 0.02).

**Table 1:** Characteristics of the patients with HF.

	All HF	non-obese (NOB) HF	obese (OB) HF	p
	(N = 16)	(N = 8)	(N = 8)	
Age, y	63 ± 9	61 ± 8	65 ± 11	0.38
**BMI, kg/m²**	29.5 ± 6.3	24.2 ± 1.9	34.8 ± 4.1	** < 0.01**
**Cardiovascular risk factors**				
Hypertensive, % (n)	31 (5)	12 (1)	50 (4)	0.28
**T2-Diabetes, % (n)**	37 (6)	0 (0)	75 (6)	**0.01**
Dyslipedemia, % (n)	56 (9)	37 (3)	75 (6)	0.31
Smoking, % (n)	12 (2)	12 (1)	12 (1)	1.00
Alcool, % (n)	12 (2)	0 (0)	25 (2)	0.47
**Heart failure etiology**				
Ischemic CM, % (n)	62 (10)	75 (6)	50 (4)	0.61
Dilated CM, % (n)	37 (6)	25 (2)	50 (4)	0.61
**Medication**				
ACEIs, % (n)	94 (15)	100 (8)	87 (7)	1.00
Beta-blockers, % (n)	94 (15)	100 (8)	87 (7)	1.00
Diuretics, % (n)	94 (15)	75 (8)	87 (7)	1.00
Vitamin K antagonists, % (n)	44 (7)	50 (4)	37 (3)	1.00
Antiplatelet agents, % (n)	56 (9)	62 (5)	50 (4)	1.00
Statines, % (n)	62 (10)	75 (6)	50 (4)	0.61
Insulin, % (n)	19 (3)	0 (0)	37 (3)	0.20
**Admission labs**				
**BNP, pmol/ml**	237 [116–1054]	976 [141–7284]	160 [26–600]	**0.05**
Creatinin, μmol/L	124 ± 10.74	122 ± 37	125 ± 48	0.89
C reactive protein, mg/L	6.5 [3.8–12.0]	10.2 [3.5–27.9]	6.2 [2.6–10.9]	0.35
Na^+^, mmol/L	137.9 ± 3.9	136.3 ± 3.9	139.5 ± 3.4	0.10
K^+^, mmol/L	4.1 ± 0.3	4.1 ± 0.3	4.2 ± 0.3	0.73
**Total Proteins, g/L**	73 ± 11	68 ± 8	79 ± 11	**0.04**
Hemoglobin, g/dl	14.3 ± 1.9	13.8 ± 2.3	14.7 ± 1.6	0.39
Hematocrit, %	42 ± 5	40 ± 6	44 ± 4	0.21
Leucocytes,x 10^9^/L	7.9 ± 2.2	6.8 ± 1.5	8.9 ± 2.5	0.06
Bilirubin, mg/L	6.0 [4.2–6.0]	6.0 [4.5–6.5]	5.0 [4.0–6.0]	0.51
**TGO (ASAT), UI**	28.0 ± 9.5	23.1 ± 3.9	34.8 ± 11.3	**0.02**
**TGP (ALAT), UI**	32.9 ± 20.3	24.7 ± 6.2	44.4 ± 28.2	**0.04**
Triglyceride, g/L (0,6–1,7)	1.0 [0.7–1.5]	1.0 [0.7–7.4]	1.5 [0.7–3.6]	0.52
LDL, g/L	0.80 [0.65–0.99]	0.85 [0.52–1.14]	0.80 [0.65–1.35]	0.77
HDL, g/L	0.40 [0.40–0.50]	0.40 [0.25–0.55]	0.50 [0.40–0.55]	0.23
Cholesterol total, g/L (1,5–2,2)	1.5 [1.4–2.0]	1.5[1.0–2.3]	1.7 [1.4–2.1]	0.56
**Admission vitals**				
MAP, mmHg	91 ± 10	87 ± 7	94 ± 10	0.45
HR, Bpm	70 ± 5.38	74 ± 21	67 ± 21	0.54
**Echocardiography**				
**LVEF, %**	33 ± 15	25 ± 7	42 ± 17	**0.02**
LVEF < 30%, % (n)	50 (8)	75 (6)	25 (2)	0.13
**NYHA class**				
I,% (n)	6 (1)	0	12 (1)	1.00
II,% (n)	68 (11)	75 (6)	62 (5)	1.00
III,% (n)	25 (4)	25 (2)	25 (2)	1.00
**Outcomes**				
**All cases mortality**	25 (5)	50 (5)	0	**0.02**

HF, heart failure; BMI, body mass index; CM, cardiomyopathy; ACEIs, angiotensin-converting enzyme inhibitors; BNP, B-type natriuretic peptide concentration; TGP (ALT), alanine aminotransferase; TGO (AST), aspartate aminotransferase; MAP, Mean Arterial Pressure; HR, Heart rate; LDL, low density lipoprotein; HDL, high density protein; LVEF, left ventricular ejection fraction; NYHA class, New York Heart Association functional classification. Results are presented as mean ± SEM or ± confidence intervals. Bold values are significant vs NOB group.

**Figure 2:**
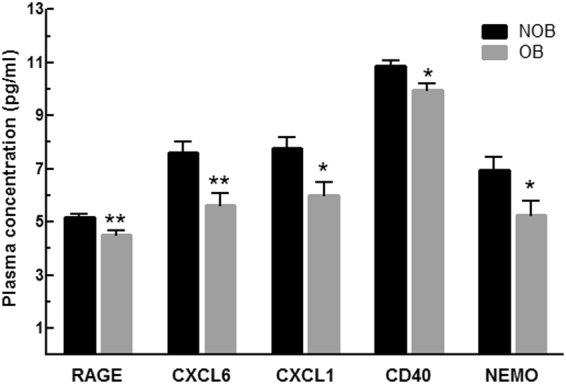
Pro-inflammatory profiling in NOB and OB patients with HF. Plasma protein levels of RAGE, CXCL6, CXCL1, CD40 and NEMO in NOB (n = 8) and OB (n = 8) HF patients. Data shown are mean ± SEM. *p < 0.05 vs NOB; **p < 0.01 vs NOB.

**Figure 3:**
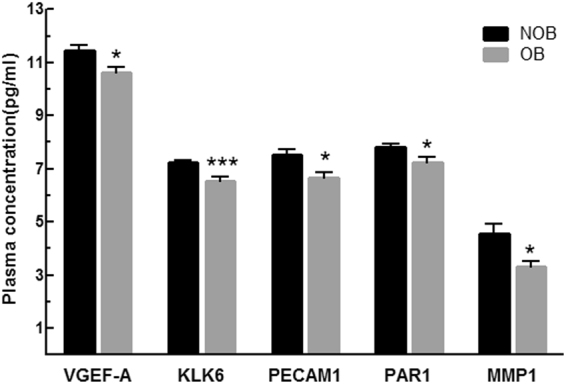
Pro-angiogenic profiling in NOB and OB patients with HF. Plasma level of VEGF-A, KLK6, PECAM1, PAR1, MMP1 in NOB (n = 8) and OB (n = 8) HF patients. Data shown are mean ± SEM. *p < 0.05 vs NOB; ***p < 0.005 vs NOB.

In order to assess the plasma protein fingerprint associated with cardio-inflammatory and metabolic status of HF patients in relation to BMI, we compared the plasma protein levels obtained by PEA immunoassay in NOB and OB patients. Plasma levels of 92 proteins were assessed simultaneously in 1 µl of human plasma and revealed distinct circulating multi-marker profile in OB and NOB HF patients. A total of 13 proteins were shown to be differentially expressed in NOB and OB HF patients. As shown in Fig. 2, OB HF patients demonstrated lower levels of proteins associated with inflammatory status (Receptor for advanced glycosylation end products (RAGE), C-X-C motif chemokine 6 (CXCL6), C-X-C motif chemokine 1 (CXCL1), CD40L receptor (CD40) and NF-kappa-B essential modulator (NEMO) as compared to NOB HF patients. Interestingly, we found lower level of circulating proteins related to angiogenesis (Vascular endothelial growth factor A (VEGF-A), Kallikrein-6 (KLK6), Platelet endothelial cell adhesion molecule (PECAM1), Proteinase-activated receptor 1 (PAR1) and Matrix metalloproteinase-1 (MMP1) in OB HF patients (Fig. 3).

**Figure 4:**
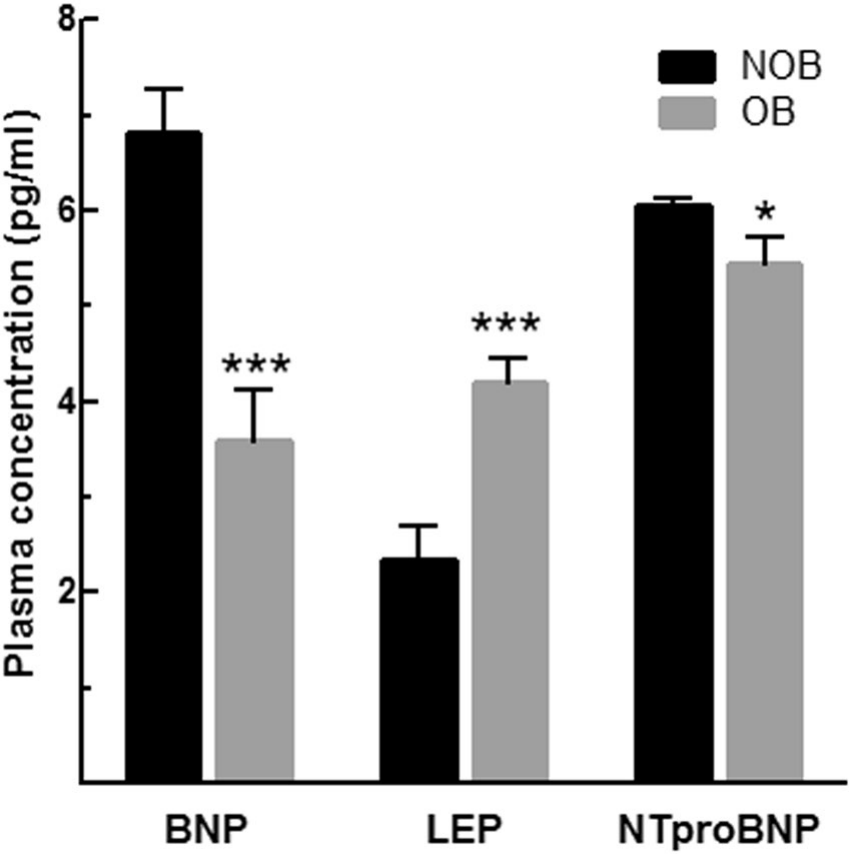
Cardio-metabolic signature of NOB and OB patients with HF. Circulating plasma level of leptin, BNP and NT-proBNP in NOB (n = 8) and OB (n = 8) HF patients. *p < 0.05 vs NOB; ***p < 0.005 vs NOB.

To investigate the plasma patterns associated with cardio-metabolic status, we compared plasma level of leptin, N-terminal pro-brain natriuretic peptide (NTproBNP) and BNP in HF patients in relation to BMI. As shown in Fig. 4, OB patients with HF had greater level of leptin and lower level of NTproBNP and BNP in plasma as compared to NOB HF subjects.

## Discussion

Despite the adverse effects of obesity on left ventricle structure and function as well as the epidemiological data showing a powerful relationship between obesity and HF prevalence, numerous clinical studies have suggested that OB patients with HF have a better prognosis than lean patients^12^. The reasons for the obesity paradox in CVD, including HF, remain unclear and are somewhat difficult to reconcile. Given the complex nature of cardiac remodeling in OB phenotype, we revealed distinct patterns of plasma proteins related to pro-inflammatory, angiogenic and metabolic status in “obesity paradox” patients with HF using a proteomics-based approach on plasma samples. These results suggest that circulating level of biomarkers in HF patients can be profoundly affected by obesity and that the multi-marker approach, reflecting various biochemical pathways simultaneously, might facilitate the transition from the current paradigms of classical clinical decision-making to the new era of a more “personalized” medicine for OB HF patients.

Changes in the adipose tissue secretory capacity during the progression of HF might be important in the obesity-HF link. Various cytokines and neuroendocrine profiles of OB patients may be protective^13^. Adipose tissue is known to produce soluble tumor necrosis factor-alpha receptors, which could have a protective effect in OB patients with both acute and chronic HF by neutralizing the adverse biological effects of tumor necrosis factor-alpha^14,15^. Studies have also demonstrated that overweight and OB patients generally have a reduced expression of circulating natriuretic peptides, which Mehra *et al*.^16^, also demonstrated in HF, potentially leading to OB patients becoming symptomatic and thus presenting earlier at less severe stages of HF. Additionally, OB patients may have an attenuated response of the angiogenic status, which may also lead to a better prognosis^17^. In addition, higher circulating lipoproteins in OB patients may bind and detoxify lipopolysaccharides that play a role in stimulating the release of pro-inflammatory cytokines, all of which may serve to protect OB HF patients^13^. Certainly, most of the HF studies that demonstrated the obesity paradox expressed body habitus by BMI alone, which did not provide for the most accurate reflection of adipose tissue.

Currently there are no specific guidelines for the diagnosis and treatment of HF in OB patients. Therefore, there is an urgent need to identify new circulating biomarkers/panels of proteins that could identify “obesity paradox” phenotype in HF patients^18^. Biomarkers in HF research have been used to provide pathophysiological insights, to aid in establishing the diagnosis, refine prognosis, guide management, and target treatment. Given the complex nature of cardiac remodeling in OB phenotype, proteomic profiling for biomarkers may have great promise in revealing optimal strategy for diagnosis and treatment of HF according to BMI. In this study, we identify a biomarker panel that has higher sensitivity and specificity for “obesity paradox” phenotype. Using comparative plasma proteomic profiling we revealed distinct patterns of pro-inflammatory, angiogenic and metabolic factors in OB patients with HF. Although relatively high sensitivity and specificity was obtained for each individual marker in our patient groups, we believe that using rather a panel of multiple biomarkers and combination with various anthropometric parameters would be an appropriate approach in the risk stratification of OB and NOB patients with HF. We also described the relationship between levels of leptin, a key regulator of lipid metabolism, and levels of BNP, an important biomarker with an established role in the diagnosis of HF patients. This study provides the first evidence that in OB HF patients, plasma leptin concentration is inversely related to BNP concentration. This is in line with our recent study demonstrating the paradox of low BNP levels and favorable clinical prognosis in obesity^19^. Numerous studies have suggested that, among patients with HF, obesity is associated with a better overall clinical prognosis and patients with more severe HF tend to have lower BMI than do age- and gender-matched control subjects^20–22^.

Traditional HF biomarkers, even BNP and NT-proBNP, did not provide additional therapeutic target possibilities. Therefore, determining specific groups of circulating proteins may lead to better prognostication and tailored treatments in specific subgroups of HF patients. In fact, several human clinical studies exploring multi-omics approaches in HF have identified unique circulating metabolic profiles that differ between patients with HF and healthy subjects, as well as between different HF stages and HF phenotypes^23^. In the context of the coexistence of HF and obesity, our study marks the first demonstration that OB and NOB patients with HF display distinct plasma protein signature in relation to mortality outcome. Larger studies are required to confirm these observations.

## Conclusions

This exploratory study revealed plasma multi-marker signatures that can distinguish OB patients from NOB patients with HF in relation to the cardio-inflammatory, angiogenic and metabolic status. These data may help to improve the diagnosis, prognostic classification and risk prediction in OB patients with HF. Future studies on larger cohorts with well-defined phenotypes as well as the longitudinal follow-up during HF progression are needed to prove the suitability of these proteins or their combinations as biomarkers for obesity-associated CVD.

## Electronic supplementary material

Supplementary Table S1
