# Supplementary material for: Differential protein profiling as a potential multi-marker approach for obese patients with heart failure: A retrospective study
Source: Sci Rep. 2018 May 21;8:7894. doi: 10.1038/s41598-018-26118-9 (PMC5962559; doi:10.1038/s41598-018-26118-9)
Supplement: Supplementary file 1 — Supplementary Table S1 [file 41598_2018_26118_MOESM1_ESM.docx]

| Title page Title: Differential protein profiling as a potential multi-marker approach for obese patients with heart failure: A retrospective study **Author list**: Andrei Timotin^1^, Mathieu Cinato^1^, Frederic Boal^1^, Sebastien Dejean^2^, Rodica Anesia^1^, Oleg Arnaut^1^, Christine Lagente^2^, Jerome Roncalli^2^, Franck Desmoulin^4^, Helene Tronchere^1*^, Oksana Kunduzova^1^  ^*#^  **Supplementary Table S1. Cardiovascular diseases related human protein biomarkers (CVD I biomarkers), Proximity Extension Assay technology, Proseek, Multiplex** | |
| --- | --- |
| 1. Adrenomedullin (AM) |  |
| 1. Agouti-related protein (AGRP) |  |
| 1. Angiopoietin-1 receptor (TIE2) |  |
| 1. Beta-nerve growth factor (Beta-NGF) |  |
| 1. Caspase-8 (CASP-8) |  |
| 1. Cathepsin D (CTSD) |  |
| 1. Cathepsin L1 (CTSL1) |  |
| 1. C-C motif chemokine 3 (CCL3) |  |
| 1. C-C motif chemokine 4 (CCL4 ) |  |
| 1. C-C motif chemokine 20 (CCL20) |  |
| 1. CD40 ligand (CD40-L) |  |
| 1. CD40L receptor (CD40) |  |
| 1. Chitinase-3-like protein 1 (CHI3L1) |  |
| 1. C-X-C motif chemokine 1 (CXCL1) |  |
| 1. C-X-C motif chemokine 6 (CXCL6) |  |
| 1. C-X-C motif chemokine 16 (CXCL16) |  |
| 1. Cystatin-B (CSTB) |  |
| 1. Dickkopf-related protein 1 (Dkk-1) |  |
| 1. Endothelial cell-specific molecule 1 (ESM-1) |  |
| 1. Eosinophil cationic protein (ECP) |  |
| 1. Epidermal growth factor (EGF) |  |
| 1. E-selectin (SELE) |  |
| 1. Fatty acid-binding protein, adipocyte (FABP4) |  |
| 1. Fibroblast growth factor 23 (FGF-23) |  |
| 1. Follistatin (FS) |  |
| 1. Fractalkine (CX3CL1 ) |  |
| 1. Galanin peptides (GAL) |  |
| 1. Galectin-3 (Gal-3) |  |
| 1. Growth hormone (GH) |  |
| 1. Growth/differentiation factor 15 (GDF-15) |  |
| 1. Heat shock 27 kDa protein (HSP 27) |  |
| 1. Pro Heparin-binding EGF-like growth factor (HB-EGF) |  |
| 1. Hepatocyte growth factor (HGF) |  |
| 1. Interleukin-1 receptor antagonist protein (IL-1ra) |  |
| 1. Interleukin-4 (IL-4) |  |
| 1. Interleukin-6 (IL-6) |  |
| 1. Interleukin-6 receptor subunit alpha (IL-6RA) |  |
| 1. Interleukin-8 (IL-8) |  |
| 1. Interleukin-16 (IL-16) |  |
| 1. Interleukin-18 (IL-18) |  |
| 1. Interleukin-27 (IL-27) |  |
| 1. Kallikrein-6 (KLK6 ) |  |
| 1. Kallikrein-11 (hK11) |  |
| 1. Lectin-like oxidized LDL receptor 1 (LOX-1) |  |
| 1. Leptin (LEP) |  |
| 1. Macrophage colony-stimulating factor 1 (CSF-1) |  |
| 1. Matrix metalloproteinase-1 (MMP-1) |  |
| 1. Matrix metalloproteinase-3 (MMP-3) |  |
| 1. Matrix metalloproteinase-7 (MMP-7) |  |
| 1. Matrix metalloproteinase-10 (MMP-10) |  |
| 1. Matrix metalloproteinase-12 (MMP-12) |  |
| 1. Melusin (ITGB1BP2) |  |
| 1. Membrane-bound aminopeptidase P (mAmP) |  |
| 1. Monocyte chemotactic protein 1 (MCP-1) |  |
| 1. Myeloperoxidase (MPO) |  |
| 1. Myoglobin (MB) |  |
| 1. Natriuretic peptides B (BNP) |  |
| 1. NF-kappa-B essential modulator (NEMO) |  |
| 1. Osteoprotegerin (OPG) |  |
| 1. Ovanrian cancer-related tumor marker CA 125 (CA125) |  |
| 1. Pappalysin-1 (PAPPA) |  |
| 1. Pentraxin-related protein PTX3 (PTX3) |  |
| 1. Placenta growth factor (PlGF) |  |
| 1. Platelet endothelial cell adhesion molecule (PECAM-1) |  |
| 1. Platelet-derived growth factor subunit B (PDGF subunit B) |  |
| 1. Prolactin (PRL) |  |
| 1. Protein S100-A12 (EN-RAGE) |  |
| 1. Proteinase-activated receptor 1 (PAR-1) |  |
| 1. Proto-oncogene tyrosine-protein kinase Src (SRC) |  |
| 1. P-selectin glycoprotein ligand 1 (PSGL-1) |  |
| 1. Receptor for advanced glycosylation end products (RAGE) |  |
| 1. Renin (REN) |  |
| 1. Resistin (RETN) |  |
| 1. SIR2-like protein (SIRT2) |  |
| 1. Spondin-1 (SPON1) |  |
| 1. ST2 protein (ST2) |  |
| 1. Stem cell factor (SCF) |  |
| 1. Thrombomodulin (TM) |  |
| 1. TIM-1 |  |
| 1. Tissue factor (TF) |  |
| 1. Tissue-type plasminogen activator (t-PA) |  |
| 1. TNF-related activation-induced cytokine (TRANCE) |  |
| 1. TNF-related apoptosis-inducing ligand (TRAIL) |  |
| 1. TNF-related apoptosis-inducing ligand receptor 2 (TRAIL-R2) |  |
| 1. Tumor necrosis factor receptor 1 (TNF-R1) |  |
| 1. Tumor necrosis factor receptor 2 (TNF-R2) |  |
| 1. Tumor necrosis factor receptor superfamily member 5 |  |
| 1. Tumor necrosis factor receptor superfamily member 6 (FAS) |  |
| 1. Tumor necrosis factor ligand superfamily member 14 (TNFRSF14) |  |
| 1. Urokinase plasminogen activator surface receptor (U-PAR) |  |
| 1. Vascular endothelial growth factor A (VEGF-A) |  |
| 1. Vascular endothelial growth factor D (VEGF-D) |  |
